# Integrating reproductive and metabolic factors for uterine fibroid risk assessment: a two-center machine learning study with SHAP interpretability

**DOI:** 10.3389/fonc.2026.1703668

**Published:** 2026-03-23

**Authors:** Yunxia Ji, Yun Shen, Yahui Wu, Ke Lei, Min Kang, Jinsheng Wang

**Affiliations:** 1 Department of Pathology, Heping Hospital Affiliated to Changzhi Medical College, Changzhi, Shanxi, China; 2 Department of Pathology, People’s Hospital of Tongling City, Tongling, Anhui, China; 3 Department of Pathology, The First Clinical College of Changzhi Medical College, Changzhi, Shanxi, China

**Keywords:** machine learning, random forest, risk prediction, SHAP interpretability, uterine fibroids

## Abstract

**Background:**

Uterine fibroids are common benign gynecological tumors that adversely affect reproductive health. This study aimed to develop a machine learning–based model to predict individualized fibroid risk in women of reproductive age.

**Methods:**

Six clinical predictors encompassing reproductive and metabolic factors were analyzed. Feature selection was performed using LASSO regression, and multiple machine learning algorithms with cross-validation were compared. The optimal model was assessed for discrimination, calibration, and clinical benefit, with SHapley Additive exPlanations (SHAP) analysis employed to enhance interpretability.

**Results:**

A total of 1,274 women were included, of whom 762 (59.8%) had uterine fibroids. Six predictors were retained, and among the classifiers tested, the Random Forest achieved the best validation performance (AUC = 0.734) with balanced accuracy and F1 score. SHAP interpretation further identified age, BMI, menarche age, parity, triglycerides, and fasting glucose as the most influential risk factors.

**Conclusion:**

An interpretable Random Forest model was established to predict uterine fibroid risk, enabling individualized risk stratification and supporting timely preventive interventions in clinical practice.

## Introduction

Uterine fibroids, also known as leiomyomas, are the most common benign tumors of the female reproductive tract, primarily affecting women of reproductive age. Epidemiological studies demonstrate that their prevalence increases with advancing age until menopause, with lifetime incidence estimates exceeding 70% in some populations, particularly among women of African descent (1, 2). Fibroids are associated with a wide range of morbidities, including heavy menstrual bleeding, pelvic pain or pressure, infertility, and adverse pregnancy outcomes, thereby constituting a major public health burden worldwide (2, 3).

The etiology of uterine fibroids is multifactorial, involving both non-modifiable and modifiable determinants. Among the non-modifiable factors, age, race, and genetic predisposition consistently emerge as strong predictors. Women of African ancestry have a higher incidence, earlier onset, and greater symptom severity compared with other ethnic groups (4). Reproductive history also influences risk: early menarche and nulliparity are associated with increased risk, likely due to prolonged lifetime estrogen exposure and increased ovulatory cycles (5, 6). Conversely, higher parity and later age at first birth appear protective, possibly through postpartum uterine remodeling and hormonal changes. In addition, metabolic and cardiovascular factors such as elevated body mass index (BMI) and hypertension have been implicated in fibroid pathogenesis, potentially via chronic inflammation, altered hemodynamics, and endocrine dysregulation (7, 8). Lifestyle and environmental exposures, including diet, alcohol intake, and physical activity, have also been investigated, although results remain heterogeneous across studies (9). Recent studies further highlight potential role of reproductive and metabolic factors, such as BMI, age at menarche, as well as parity in predicting the risk of uterine fibroids (5, 6).

Despite growing evidence on epidemiological and clinical risk factors, robust predictive models integrating multidimensional indicators are scarce. Existing approaches often focus on limited clinical variables or lack rigorous validation (10). To date, no widely accepted model has been established to predict the first onset of uterine fibroids in women of reproductive age by combining demographic, reproductive, and metabolic parameters.

Several previous studies have attempted to develop predictive models for uterine fibroids or related clinical outcomes. For example, Song et al. developed and validated a nomogram-based model to predict recurrence risk after myomectomy, demonstrating the feasibility of individualized risk assessment in uterine leiomyoma (10). However, existing models have mainly focused on postoperative recurrence rather than the primary onset of fibroids, relied largely on conventional statistical approaches, or incorporated a limited number of clinical variables (11, 12). Moreover, machine learning–based models that integrate multidimensional reproductive and metabolic factors for individualized primary risk prediction of uterine fibroids remain scarce. Therefore, there is still a substantial need for robust and interpretable predictive models to support early identification and targeted prevention strategies in women of reproductive age.

To address these limitations, we developed a comprehensive and interpretable machine learning–based prediction model for the initial occurrence of uterine fibroids in women of reproductive age by integrating clinical, metabolic, and reproductive factors. Least absolute shrinkage and selection operator (LASSO) regression was applied for feature selection, followed by the construction and comparison of multiple machine learning algorithms to generate individualized risk estimates. Furthermore, Shapley additive explanations (SHAP) were employed to enhance model transparency and clinical interpretability.

## Materials and methods

### Study design and population

**Figure 1:**
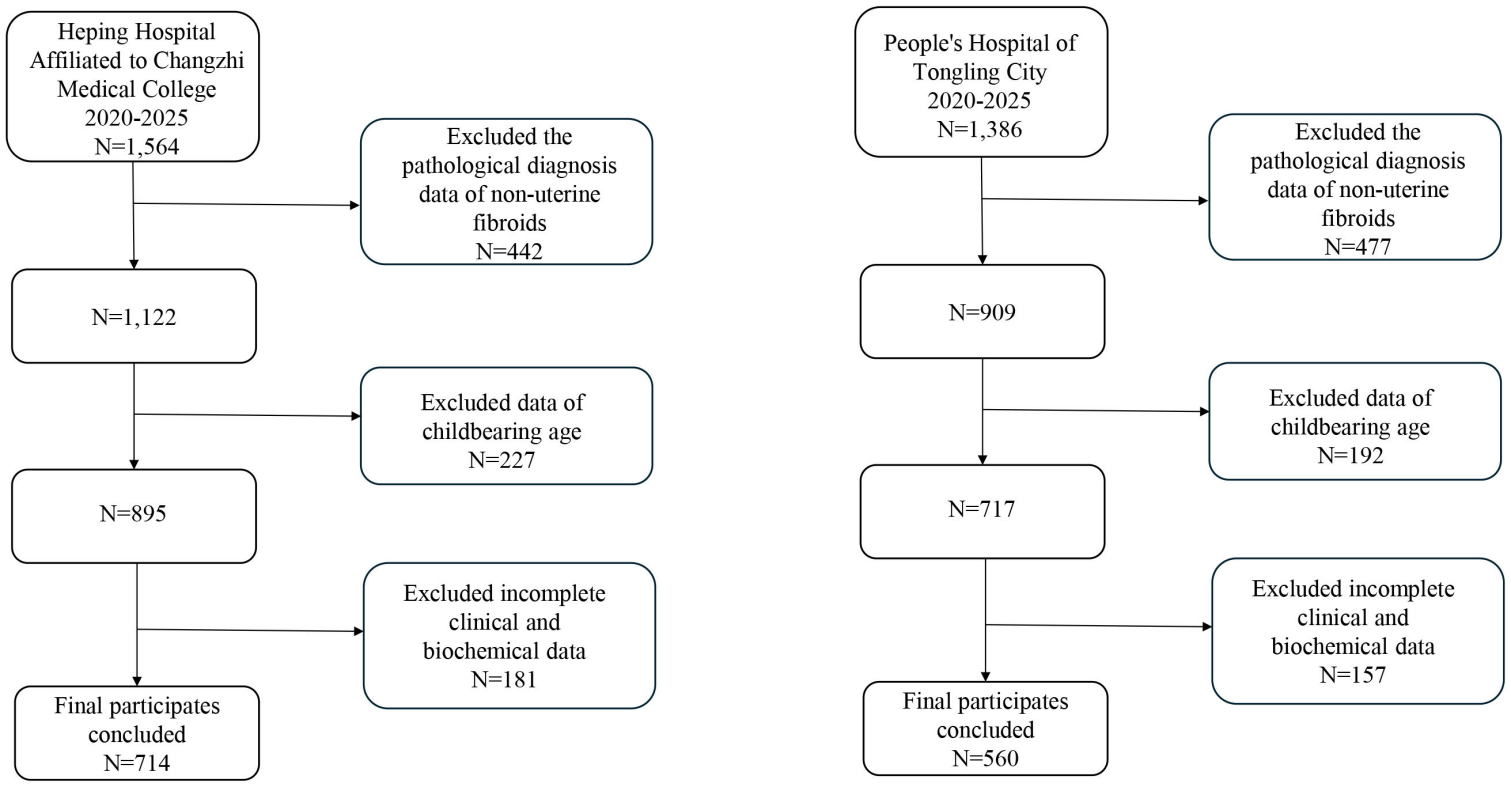
Flowchart of patient inclusion and exclusion. The diagram illustrates the selection process of study participants. From two tertiary medical centers, patients were screened according to predefined eligibility criteria. After excluding those with non-pathological diagnoses, outside reproductive age, or incomplete records, 1,274 women were retained.

This retrospective study included women histologically diagnosed with uterine fibroids at two tertiary medical centers in China. All diagnoses were confirmed histopathologically, which is the gold standard for fibroid identification (1, 2). Reproductive age was defined as 15 to 49 years, consistent with established guidelines for women of childbearing age. Participants were selected after excluding those with non-pathological findings, those outside the reproductive age range, and those with missing key clinical information required for defining the outcome or major predictors. A total of 1,274 eligible women were retained for analysis. Data were collected from Heping Hospital (Changzhi Medical College, Shanxi Province) and Tongling People’s Hospital (Anhui Province). The pooled cohort was randomly divided into training (70%) and internal validation (30%) sets (**Figure 1**). Missing data were addressed through multiple imputation, a method that preserves sample size and minimizes bias. Five imputations were performed using a fixed random seed to ensure reproducibility, thus no data were excluded during the analysis.

### Outcome and predictors

The outcome variable was the presence of uterine fibroids, classified as a binary variable (absence vs. presence). Based on prior literature and clinical guidelines (3, 5, 6, 10, 13), a broad spectrum of candidate predictors was included, encompassing demographic and metabolic characteristics (age, body mass index, marital status, educational level, lipid profile, and fasting glucose), reproductive factors (age at menarche, menstrual irregularities, miscarriage history, parity, and contraceptive use), inflammatory and hematological indicators (neutrophil-to-lymphocyte ratio, hemoglobin concentration, CA125, and CA199 levels), medical and family history (pelvic inflammatory disease, cervicitis, family history of uterine fibroids, diabetes, cardiovascular disease, and hypertension), as well as lifestyle factors (smoking, alcohol intake, and physical activity). These predictors were selected because they represent clinically relevant and biologically plausible determinants of uterine fibroid development and have been reported in previous epidemiological and mechanistic studies (8, 14).

### Data preprocessing

To minimize bias from incomplete records, missing values were handled using multiple imputation, which preserves sample size and reduces distortion from case-wise deletion (15). Five imputations were performed with a fixed random seed to ensure reproducibility. Continuous variables were subsequently standardized by Z-score normalization to place predictors on a comparable scale, improve numerical stability, and reduce the risk of overfitting. Following preprocessing, the dataset was stratified and randomly divided into training (70%) and validation (30%) sets to maintain the outcome distribution and enhance model generalizability (16). The training set was used to construct and optimize the prediction models, while the validation set was reserved as an independent internal cohort to assess discrimination, calibration, and clinical utility, thereby minimizing overfitting and ensuring robust model evaluation. This approach ensured that model development accurately reflects the underlying cohort structure while reducing the likelihood of biased parameter estimation. As the outcome distribution was relatively balanced, no synthetic resampling methods such as SMOTE were necessary. Model training and internal validation were performed exclusively within the training set, while the internal validation set was reserved for unbiased evaluation of predictive performance and clinical applicability.

**Figure 2:**
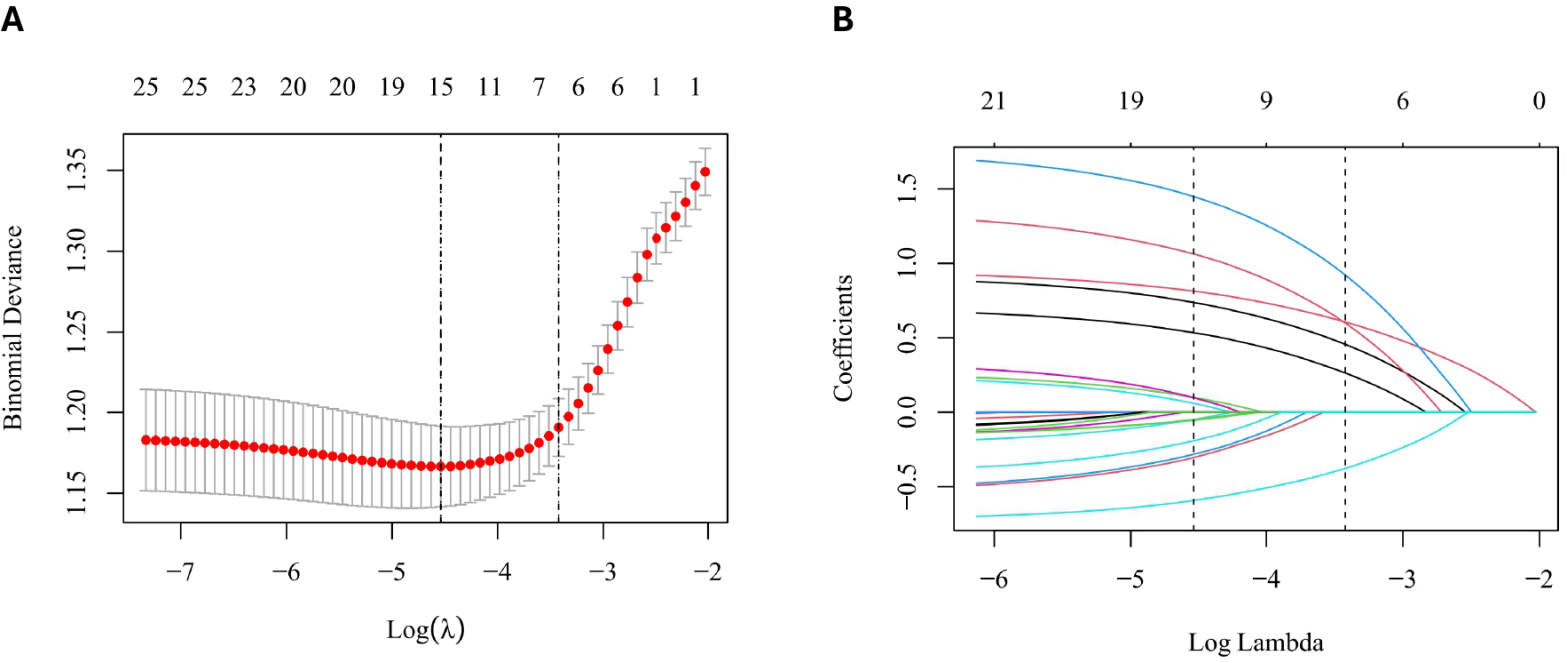
LASSO feature selection. **(A)** Coefficient paths of candidate variables across log(λ). **(B)** 10-fold cross-validation curve with λ_min_ and λ_1se_ indicated. Six predictors—age, body mass index (BMI), menarche age, parity, triglycerides (TG), and fasting plasma glucose (FPG)—were retained at λ_1se_.

To construct the prediction model, we applied LASSO regression with binomial family and L1 regularization to identify the most informative predictors (17). Numeric variables were standardized using Z-score normalization, categorical variables were converted into factors, and a fixed random seed was applied to ensure reproducibility. The model was trained with 1000 candidate lambda values, and 10-fold cross-validation determined the optimal penalty. Using the one-standard-error criterion (λ_1se_), six predictors—age, BMI, menarche age, parity, triglycerides (TG), and fasting plasma glucose (FPG)—were retained. These features were then incorporated into seven machine learning algorithms: Logistic Regression (LR), Decision Tree (DT), Support Vector Machine (SVM), Artificial Neural Network (ANN), LightGBM, XGBoost, and Random Forest (RF) (**Figures 2A, B**). Model development was conducted on the training set using 5-fold cross-validation with resampling, with the internal validation set reserved for hyperparameter tuning. Model performance and clinical utility were rigorously evaluated through three complementary approaches: calibration curves for assessing concordance between predicted and observed outcomes, decision curve analysis (DCA) for quantifying clinical net benefit, and receiver operating characteristic (ROC) curves for characterizing the sensitivity–specificity trade-off. Furthermore, metrics including AUC, accuracy, precision, sensitivity, specificity, and F1 score were calculated to provide a comprehensive assessment of discrimination and robustness.

### Machine learning algorithms

Seven algorithms were implemented: LR, DT, RF, SVM, ANN, XGBoost, and LightGBM. Hyperparameter optimization was conducted using grid search with 5-fold cross-validation. LR served as the baseline model (18). DT provides interpretable hierarchical splits (19), while RF reduces variance by aggregating multiple trees (20). XGBoost applies gradient boosting to sequentially refine weak learners (21), and LightGBM accelerates training with optimized tree growth (22). SVM constructs maximal-margin decision boundaries in high-dimensional spaces (23), whereas ANN captures complex nonlinear patterns (24). The optimal hyperparameters identified were: DT (ccp_alpha = 0.0, max_depth = 5, max_features = “sqrt”, min_samples_split = 20); RF (n_estimators = 250, max_features = 2); XGBoost (learning_rate = 0.01, max_depth = 3, n_estimators = 200, subsample = 0.6); LightGBM (colsample_bytree = 0.6, lambda_l2 = 0.1, learning_rate = 0.01, max_depth = 6, min_data_in_leaf = 30, n_estimators = 200, num_leaves = 31, subsample = 0.6); SVM (C = 0.1, degree = 2, gamma = “scale”, kernel = “linear”); ANN (activation = “logistic”, hidden_layer_sizes = (100)),. These configurations substantially improved predictive accuracy and robustness.

### Model evaluation

Model performance and clinical utility were evaluated using calibration curves to assess the concordance between predicted and observed outcomes, DCA to quantify the clinical net benefit across various thresholds, and ROC curves to characterize sensitivity–specificity trade-offs. Furthermore, discriminative ability was comprehensively quantified using the AUC, accuracy, precision, sensitivity, specificity, and F1 score.

### SHAP interpretability analysis

Model interpretability was evaluated using Shapley additive explanations (SHAP), a game theory–based method that attributes the marginal contribution of each predictor to the model output (25). Positive SHAP values indicated increased fibroid risk, whereas negative values suggested protective effects. Both global importance ranking and individual-level contributions were analyzed, enhancing transparency and clinical interpretability.

### Statistical analysis

All analyses were conducted using R version 4.4.3 (R Foundation for Statistical Computing) for data preprocessing and Python version 3.10.4 (Python Software Foundation) for model training and interpretation. Continuous variables were summarized as mean ± standard deviation or median (interquartile range), depending on distribution, and compared using Student’s t-test or the Wilcoxon rank-sum test. Categorical variables were reported as counts and percentages, with group differences evaluated using chi-square or Fisher’s exact test as appropriate. A two-tailed P < 0.05 was considered statistically significant.

## Result

### Patient characteristics

**Table 1** presents the baseline characteristics of the study cohort (n = 1,274). Significant differences (p < 0.05) were observed between women with and without uterine fibroids. The age distribution revealed a higher proportion of women aged 34–49 years in the uterine fibroid group (71.26%) compared to the non-uterine fibroid group (55.47%, p < 0.001). We observed a much higher proportion of overweight/obese individuals in the uterine fibroid group (56.43%) compared to the healthy control group (31.64%). Patients with uterine fibroids had a lower age at menarche, with 69.14% of healthy women having menarche at age ≥13 years, compared to 52.23% in the uterine fibroid group (p < 0.001). This finding aligns with previous studies that identify earlier menarche as a risk factor for fibroid development. In addition, patients with uterine fibroids demonstrated a lower proportion of multiparity when compared with healthy controls (p < 0.001). Elevated triglyceride levels (133.15 mg/dl vs. 125.80 mg/dl, p < 0.001) and fasting plasma glucose (5.20 mmol/L vs. 5.00 mmol/L, p < 0.001) were higher in the uterine fibroid group. Notably, alcohol consumption was lower in the uterine fibroid group (6.56% vs. 10.55%, p = 0.015). These findings highlight the significant roles of metabolic, reproductive, and lifestyle factors in the development of uterine fibroids.

**Table 1:** Baseline data of all included patients.

Variables	Overall (N = 1,274)	Non- uterine fibroid (N = 512)	Uterine fibroids (N = 762)	*p*
**Age, [n (%)]**				**<0.001**
15-33	447 (35.09)	228 (44.53)	219 (28.74)	
34-49	827 (64.91)	284 (55.47)	543 (71.26)	
**BMI, [n (%)]**				**<0.001**
Normal weight	682 (53.53)	350 (68.36)	332 (43.57)	
Overweight	395 (31.00)	127 (24.80)	268 (35.17)	
Obese	197 (15.46)	35 (6.84)	162 (21.26)	
**Marital status, [n (%)]**				0.441
Married/ Living with Partner	906 (71.11)	354 (69.14)	552 (72.44)	
Never married	248 (19.47)	107 (20.90)	141 (18.50)	
Widowed/ Divorced/ Separated	120 (9.42)	51 (9.96)	69 (9.06)	
**Education level, [n (%)]**				0.824
Less than high school	257 (20.17)	106 (20.70)	151 (19.82)	
High school or above high school	960 (75.35)	385 (75.20)	575 (75.46)	
Others	57 (4.47)	21 (4.10)	36 (4.72)	
**Menarche age, [n (%)]**				**<0.001**
<13	522 (40.97)	158 (30.86)	364 (47.77)	
≥ 13	752 (59.03)	354 (69.14)	398 (52.23)	
**Menstrual disorder, [n (%)]**				0.818
No	966 (75.82)	386 (75.39)	580 (76.12)	
Yes	308 (24.18)	126 (24.61)	182 (23.88)	
**Abortion history, [n (%)]**				0.921
No	773 (60.68)	312 (60.94)	461 (60.50)	
Yes	501 (39.32)	200 (39.06)	301 (39.50)	
**Parity, [n (%)]**				**<0.001**
≤ 2	621 (48.74)	285 (55.66)	336 (44.09)	
>2	653 (51.26)	227 (44.34)	426 (55.91)	
**Contraceptive use, [n (%)]**				0.750
No	1014 (79.59)	408 (79.69)	606 (79.53)	
Yes	260 (20.41)	104 (20.31)	156 (20.47)	
Pelvic inflame, [n (%)]				
No	1104 (86.66)	453 (88.48)	651 (85.43)	0.138
Yes	170 (13.34)	59 (11.52)	111 (14.57)	
Cervicitis, [n (%)]				
No	1013 (79.51)	411 (80.27)	602 (79.00)	0.631
Yes	261 (20.49)	101 (19.73)	160 (21.00)	
Family history, [n (%)]				
No	1066 (83.67)	427 (83.40)	639 (83.86)	0.888
Yes	208 (16.33)	85 (16.60)	123 (16.14)	
Diabetes, [n (%)]				
No	1140 (89.48)	456 (89.06)	684 (89.76)	0.759
Yes	134 (10.52)	56 (10.94)	78 (10.24)	
CVD, [n (%)]				
No	1121 (87.99)	455 (88.87)	666 (87.40)	0.483
Yes	153 (12.01)	57 (11.13)	96 (12.60)	
Hypertension, [n (%)]				
No	1087 (85.32)	434 (84.77)	653 (85.70)	0.705
Yes	187 (14.68)	78 (15.23)	109 (14.30)	
Smoking, [n (%)]				
No	1144 (89.80)	462 (90.23)	682 (89.50)	0.742
Yes	130 (10.20)	50 (9.77)	80 (10.50)	
**Alcohol taking, [n (%)]**				**0.015**
No	1170 (91.84)	458 (89.45)	712 (93.44)	
Yes	104 (8.16)	54 (10.55)	50 (6.56)	
**Exercise time, [n (%)]**				0.173
<150 min	816 (64.05)	316 (61.72)	500 (65.62)	
≥150 min	458 (35.95)	196 (38.28)	262 (34.38)	
**TG (mg/dl)**	129.95 [115.90, 145.00]	125.80 [111.88, 138.02]	133.15 [119.50, 147.50]	**<0.001**
**TC (mg/dl)**	189.40 [178.22, 201.55]	188.50 [176.88, 201.45]	189.80 [178.93, 201.55]	0.277
**LDL_C (mg/dl)**	120.05 [110.70, 129.40]	118.95 [110.68, 128.40]	121.10 [110.70, 129.80]	0.119
**HDL_C (mg/dl)**	54.85 [49.10, 60.10]	54.90 [49.27, 60.40]	54.80 [49.02, 59.90]	0.420
**FPG (mmol/L)**	5.10 [4.80, 5.40]	5.00 [4.80, 5.32]	5.20 [4.90, 5.40]	**<0.001**
**NLR**	2.17 [1.87, 2.47]	2.16 [1.89, 2.47]	2.17 [1.86, 2.47]	0.893
**Hb (g/L)**	125.20 [119.70, 130.30]	125.05 [119.60, 130.33]	125.20 [119.82, 130.30]	0.919
**CA125 (U/mL)**	20.10 [17.70, 22.40]	20.20 [17.80, 22.40]	20.00 [17.60, 22.40]	0.510
**CA199 (U/mL)**	15.00 [13.00, 16.80]	15.10 [13.20, 17.10]	14.90 [12.90, 16.80]	0.155

Bold values represent the variables with a statistically significant effect (p < 0.05).

### Multimodel integrated analysis for classification

Based on the performance results from both the training and internal validation sets, as well as evaluation using various performance metrics, RF emerged as the most optimal model for predicting uterine fibroid risk. In the training set, RF achieved the highest AUC of 0.839 (95% CI: 0.815–0.864), an accuracy of 75.14%, and a sensitivity of 85.96%, demonstrating strong performance in detecting true positives. Although the RF model showed good performance in the internal validation set (AUC: 0.733, accuracy: 66.67%), its specificity slightly dropped to 47%, but it still performed satisfactorily in detecting uterine fibroids. Known for its robustness against overfitting, the RF model effectively handles both linear and non-linear relationships, contributing to its stability across different datasets (**Tables 2**, **3**).

**Table 2:** Performance metrics of machine learning models on the training set.

Model	AUC	Accuracy	Precision	Sensitivity	Specificity	F1 score
Logistic	0.76	0.71	0.73	0.81	0.55	0.77
Decision Tree	0.77	0.72	0.74	0.82	0.58	0.78
Random Forest	0.84	0.75	0.76	0.86	0.59	0.81
XGBoost	0.80	0.71	0.72	0.86	0.50	0.78
LightGBM	0.84	0.76	0.76	0.88	0.58	0.81
SVM	0.76	0.70	0.72	0.81	0.54	0.76
ANN	0.76	0.70	0.71	0.82	0.51	0.76

**Table 3:** Performance metrics of machine learning models on the internal validation set.

Model	AUC	Accuracy	Precision	Sensitivity	Specificity	F1 score
Logistic	0.76	0.68	0.71	0.77	0.54	0.74
Decision Tree	0.71	0.68	0.72	0.76	0.56	0.74
Random Forest	0.73	0.67	0.69	0.81	0.47	0.74
XGBoost	0.74	0.66	0.68	0.80	0.45	0.74
LightGBM	0.73	0.65	0.68	0.80	0.44	0.73
SVM	0.76	0.67	0.70	0.76	0.52	0.73
ANN	0.76	0.68	0.71	0.80	0.51	0.75

**Figure 3:**
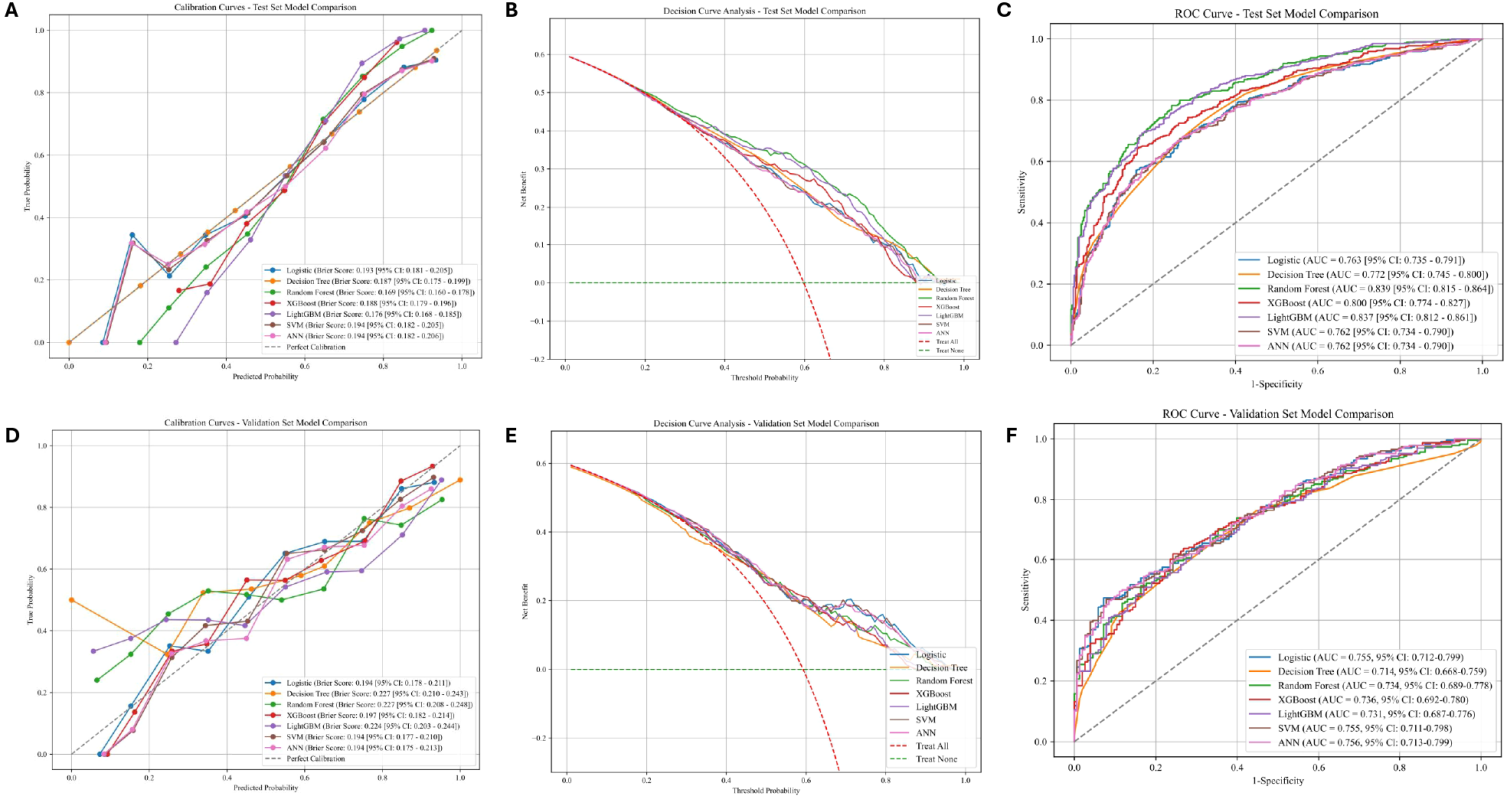
Performance comparison of machine learning models. **(A)** Calibration Curve for the training Set. **(B)** Decision Curve Analysis for the training Set. **(C)** ROC Curves for the training Set. **(D)** Calibration Curve for the internal validation set. **(E)** Decision Curve Analysis for the internal validation set. **(F)** ROC Curves for the internal validation set.

In addition, when incorporating calibration curves, decision curve analysis (DCA), and ROC curves, RF demonstrated the best balance between sensitivity and specificity, further reinforcing its generalizability and predictive power. DCA indicated that the RF model provides net clinical benefit across various threshold probabilities. This suggests that integrating the model into clinical decision-making could improve patient management by prioritizing high-risk individuals for early intervention. While other models, such as XGBoost and LightGBM, also performed well, particularly with high AUC values in both the training and internal validation sets, RF consistently provided the most stable results across different evaluation metrics. Therefore, based on its overall performance, consistency, and robustness, RF was selected as the most optimal model for predicting uterine fibroid risk (**Figure 3**).

### Interpretability and application of the model

**Figure 4:**
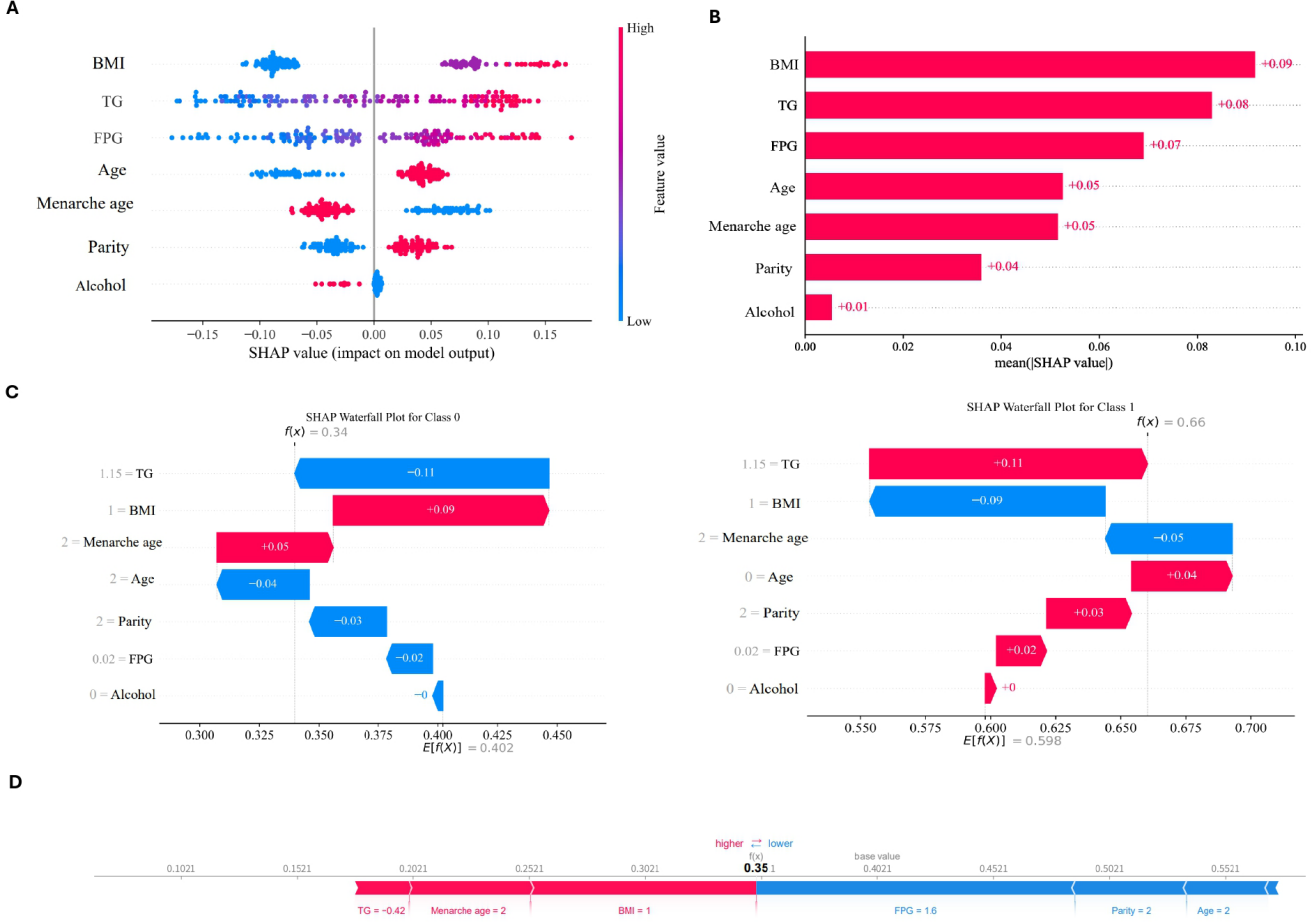
SHAP of the model. **(A)** SHAP summary showing feature contributions, with red and blue dots representing higher and lower values, respectively. **(B)** Logistic regression feature importance ranking. **(C)** Waterfall plot illustrating the combined effect of variables for a single case. **(D)** Force plot depicting individual-level risk, highlighting the balance between risk-enhancing (e.g., TG, FPG) and protective factors.

**Figure 4** presents the SHAP-based interpretation of the RF model, offering both global and individual perspectives on predictors of uterine fibroid risk. In the swarm plot (**Figure 4A**), age and BMI showed the widest spread of SHAP values, suggesting they exert the strongest influence on model predictions. In addition, older age and higher BMI were associated with an increased risk of fibroids. The importance ranking plot (**Figure 4B**) confirmed these findings, with age, BMI, menarche age, parity, triglycerides (TG), and fasting plasma glucose (FPG) identified as the six most relevant contributors. The waterfall plot (**Figure 4C**) illustrates how these variables act synergistically for an individual case: for instance, older age and higher BMI shift the predicted risk upward, whereas later menarche and higher parity mitigate this risk. The force plot (**Figure 4D**) provides a more intuitive visualization at the individual level, highlighting the balance between risk-increasing (e.g., metabolic factors such as TG and FPG) and protective factors. Collectively, these results not only validate the predictive robustness of the model but also underscore clinically recognized risk pathways of uterine fibroids, integrating reproductive history with metabolic health.

## Discussion

In this study, we constructed multiple machine learning models to predict the risk of uterine fibroids using demographic, reproductive, and metabolic features. Through rigorous feature selection and internal validation, the RF model was identified as the most effective classifier, achieving superior discrimination, calibration, and clinical utility compared with other algorithms. SHAP-based interpretation further confirmed that age, BMI, age at menarche, parity, triglycerides, and fasting plasma glucose were the most influential predictors, consistent with established biological and epidemiological evidence (26–30).

The epidemiology and pathophysiology of uterine fibroids have been extensively studied, providing a solid scientific foundation for this work. Fibroid growth was known to be hormone-dependent, with estrogen and progesterone playing key roles in tumor proliferation and extracellular matrix accumulation (13, 31). Genetic studies have identified recurrent MED12 mutations and HMGA2 rearrangements as significant factors in fibroid development (32, 33). Epidemiological evidence suggests that increasing age until menopause, early menarche, and nulliparity significantly increase the risk of fibroids (34–36), while higher parity is protective, likely due to postpartum uterine remodeling (37). Additionally, metabolic and cardiovascular factors such as elevated BMI and hypertension have been linked to the presence of fibroid risk (38–40). A large cohort study further demonstrated that elevated triglycerides and impaired glucose metabolism independently raise fibroid risk. These findings supported the integration of both reproductive and metabolic factors in predictive models; this was consistent with our RF model, which successfully captured the nonlinear interactions among these risk factors to improve prediction accuracy (40–42).

The performance of the models was compared using point estimates of AUC and 95% confidence intervals. Statistical significance was evaluated using paired t-tests, revealing that RF outperformed other models in AUC (0.734) and balanced accuracy. However, while the RF model showed moderate sensitivity, its specificity was lower in the internal validation set, leading to a higher number of false positives.

Although parity was traditionally considered a protective factor against uterine fibroid development, our study observed a higher proportion of multiparity among women with fibroids. This apparent discrepancy may be attributed to detection bias and residual confounding. Multiparous women tended to undergo more frequent gynecological examinations and imaging surveillance, which may have increased the likelihood of incidental fibroid detection. Moreover, parity is closely correlated with cumulative hormonal exposure and age, both of which are established contributors to fibroid development, potentially resulting in residual confounding. Prior longitudinal evidence suggested that pregnancy-related uterine remodeling might lead to fibroid regression, supporting the protective role of parity (43). In addition, comprehensive epidemiological reviews have highlighted the complex and sometimes conflicting associations between reproductive history and fibroid risk (2). Therefore, our findings should be interpreted with caution, and prospective cohort studies are required to further clarify the causal relationship between parity and fibroid onset.

Our study has several strengths, including its methodological rigor and interpretability. The use of LASSO regression ensured that only the most informative predictors were retained, minimizing overfitting while maintaining generalizability. Furthermore, RF, an ensemble learning algorithm, demonstrated superior ability to handle complex nonlinear relationships and variable interactions compared to linear models. SHAP analysis provided transparent explanations of the model’s predictions, enhancing clinical interpretability and reinforcing the biological plausibility of the identified risk factors. By combining traditional risk factors with advanced machine learning, our model bridges the gap between epidemiological knowledge and clinical practice.

Despite its strengths, this study has several limitations. The model was developed and internally validated using a random split of the same dataset, and further external validation in independent, multi-center cohorts is required to confirm its generalizability. Additionally, some potential predictors, such as hormonal assays, imaging data, and genetic information, were not available for this study, though their inclusion may enhance the model’s predictive power. The retrospective nature of the study also limits our ability to assess dynamic changes in risk over time. Although internal validation supports the model’s reliability, external validation across independent cohorts is essential. Future research should focus on testing the model in different clinical settings to further evaluate its performance. Recent advancements in machine learning optimization and explainable AI have introduced new techniques that could further improve the clinical applicability and accuracy of predictive models. Integrating these approaches may enhance the precision and interpretability of our fibroid prediction model (44–46).

## Conclusion

In conclusion, this study demonstrates that a RF–based machine learning model incorporating reproductive and metabolic variables can accurately predict uterine fibroid risk. The integration of SHAP analysis enhances clinical transparency and individualized risk stratification, providing a promising tool for early identification and targeted preventive strategies in gynecological practice.

## Data Availability

The raw data supporting the conclusions of this article will be made available by the authors, without undue reservation.
